# Recurrent Chest Infections to Chest Wall Swelling: Empyema Necessitans as an Unusual Presentation of Tuberculosis

**DOI:** 10.7759/cureus.118227

**Published:** 2026-10-20

**Authors:** May Htet Naing, Jinsha Khan Basheer, Muhammad Ziaullah Khan, Abdul Muktadir Shafi

**Affiliations:** 1 General Internal Medicine, Queen Elizabeth Hospital Birmingham, Birmingham, GBR; 2 Acute Medicine, Queen Elizabeth Hospital Birmingham, Birmingham, GBR; 3 Respiratory Medicine, Queen Elizabeth Hospital Birmingham, Birmingham, GBR; 4 Diabetes and Endocrinology, Queen Elizabeth Hospital Birmingham, Birmingham, GBR

**Keywords:** chest wall swelling, empyema necessitans, mycobacterium tuberculosis, pleural infection, pulmonary tb (ptb)

## Abstract

Empyema necessitans is a rare complication of pleural infection in which infected pleural fluid extends through the parietal pleura into the surrounding soft tissues. We report the case of a 32-year-old man of Indian origin living in the United Kingdom who presented with worsening right-sided pleuritic chest pain, several months of night sweats, and a persistent dry cough. Five months earlier, he had been treated for right-sided pneumonia complicated by a pleural effusion, with persistent radiographic abnormalities on follow-up. Examination revealed reduced right-sided air entry and a tender, fluctuant swelling over the right posterior chest wall. Contrast-enhanced computed tomography demonstrated a posterior pleural collection extending through the chest wall into the adjacent paraspinal musculature, consistent with empyema necessitans. Ultrasound-guided aspiration yielded frank purulent fluid. Routine bacterial, anaerobic, and fungal cultures were negative. Acid-fast bacilli were not seen on initial microscopy; however, mycobacterial culture became positive after seven days, and polymerase chain reaction (PCR) testing of the pleural fluid detected *Mycobacterium tuberculosis* complex. Whole-genome sequencing subsequently identified a lineage 1 East-African-Indian-Ocean isolate with predicted susceptibility to first-line antituberculous agents. Four-drug antituberculous therapy was commenced, with subsequent clinical and radiological improvement. This case highlights chest wall swelling as an unusual presentation of tuberculosis and emphasizes the importance of cross-sectional imaging and targeted mycobacterial testing in establishing the diagnosis.

## Introduction

Empyema necessitans (EN) is a rare complication of pleural infection characterized by extension of infected pleural fluid through the parietal pleura into the surrounding extrapleural soft tissues, most commonly the chest wall [1,2]. Its incidence has declined considerably with the advent of effective antimicrobial therapy and earlier management of pleural infections [1]. *Mycobacterium tuberculosis* and *Actinomyces* species have historically been recognized as important causative organisms, although other pathogens, including *Staphylococcus aureus*, have also been implicated [1].

Tuberculous EN may follow a chronic and indolent course, with non-specific respiratory and constitutional symptoms that can delay recognition of the underlying disease [2,3]. The development of a chest wall swelling may therefore provide an important clinical clue to extension of an underlying pleural infection. Computed tomography (CT) plays a key role in establishing the diagnosis by demonstrating continuity between the pleural collection and the extrapleural soft-tissue component and defining involvement of adjacent structures [2,4].

We report a case of tuberculous EN in a 32-year-old man with a previous episode of presumed right-sided pneumonia complicated by pleural effusion and persistent radiographic abnormalities, who subsequently developed a posterior chest wall swelling. This case highlights the diagnostic challenges associated with the indolent presentation of tuberculous EN and the importance of considering tuberculosis in patients with persistent pleuropulmonary abnormalities and an evolving chest wall mass.

## Case presentation

A 32-year-old man of Indian origin, living in the United Kingdom since 2021, presented with worsening right-sided pleuritic chest pain on a background of intermittent chest pain for approximately two years. The pain had become significantly more severe over the preceding three weeks and was described as sharp and exacerbated by deep inspiration and coughing. He also reported several months of night sweats and a persistent dry cough. There was no associated dyspnea, hemoptysis, recent travel, known tuberculosis exposure, smoking history, alcohol use, or recreational drug use. His medical history was notable only for well-controlled asthma.

Five months before the current presentation, he had been admitted with right-sided pneumonia complicated by a pleural effusion and improved following antibiotic therapy. Follow-up chest radiography, however, demonstrated persistent abnormalities, including right hilar prominence and a right upper lobe pulmonary nodule, prompting respiratory referral and investigation for possible tuberculosis.

On admission, the patient was hemodynamically stable, with a blood pressure of 115/83 mmHg, heart rate of 86 beats/min, respiratory rate of 17 breaths/min, oxygen saturation of 98% on room air, and temperature of 36°C. He appeared comfortable at rest and was not in respiratory distress. Respiratory examination demonstrated mildly reduced right-sided chest expansion and reduced air entry over the right hemithorax, with dullness to percussion over the affected area. A tender, fluctuant soft-tissue swelling was noted over the right posterior chest wall in the subscapular region, without overlying erythema, ulceration, or discharge (Figure 1).

**Figure 1:**
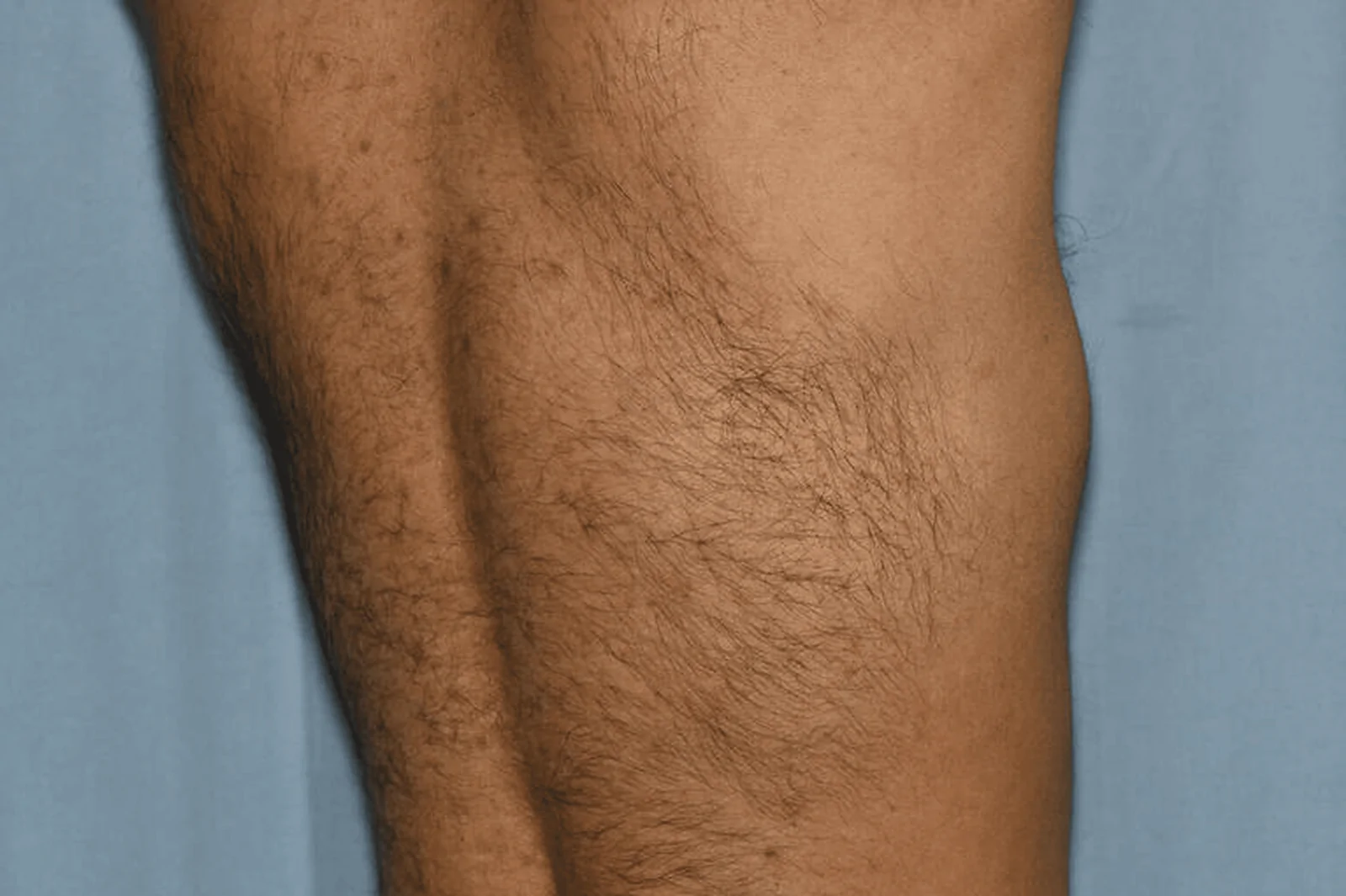
Right posterior chest wall swelling at presentation Clinical photograph demonstrating a localized swelling over the right posterior chest wall associated with empyema necessitans.

There was no peripheral lymphadenopathy, digital clubbing, peripheral edema, hepatosplenomegaly, or other significant systemic abnormality. Initial laboratory investigations demonstrated an elevated C-reactive protein level and mild microcytosis without anemia (Table 1).

**Table 1:** Laboratory parameters at admission Initial laboratory investigations demonstrated an elevated C-reactive protein level and mild microcytosis without anemia. Hemoglobin, white blood cell count, platelet count, renal function, and liver biochemical parameters were otherwise within reference ranges.

Parameter	Result	Unit	Reference range
Hemoglobin	137	g/L	130-180
Mean corpuscular volume (MCV)	79.9	fL	80-100
White blood cells	8.05	x10⁹/L	4.0-11.0
Platelets	334	x10⁹/L	150-450
Urea	3.5	mmol/L	2.5-7.8
Creatinine	82	µmol/L	60-110
Estimated glomerular filtration rate (eGFR)	>90	mL/min/1.73 m²	>60
Sodium	136	mmol/L	133-145
Potassium	4.1	mmol/L	3.5-5.1
Total bilirubin	6	µmol/L	3-21
Aspartate aminotransferase (AST)	29	U/L	11-34
Alanine aminotransferase (ALT)	37	U/L	0-55
C-reactive protein (CRP)	30	mg/L	0.0-5.0

Screening for HIV, hepatitis B, and hepatitis C was negative. Serial chest radiographs demonstrated resolution of the previously identified pleural effusion but persistent right-sided abnormalities, including right hilar fullness, a right upper lobe pulmonary nodule, and progressive parenchymal opacification. Contrast-enhanced CT of the thorax, abdomen, and pelvis demonstrated a right posterior pleural collection extending through the chest wall into the adjacent paraspinal musculature, consistent with EN (Figures 2A-2C).

**Figure 2:**
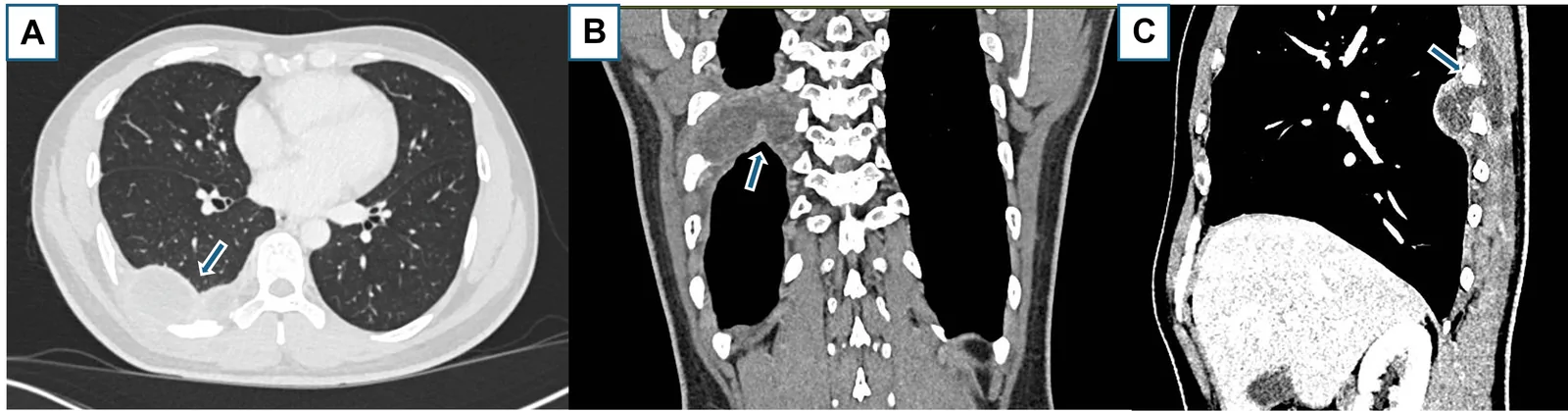
Contrast-enhanced CT of the thorax demonstrating empyema necessitans Contrast-enhanced computed tomography (CT) of the thorax in (A) axial, (B) coronal, and (C) sagittal views, demonstrating a right posterior pleural collection with extension through the chest wall into the adjacent paraspinal soft tissues (arrows), consistent with empyema necessitans.

Associated periosteal reaction of the adjacent ribs suggested chronic local inflammatory involvement, although underlying tuberculous osteomyelitis could not be excluded without further osseous evaluation. No significant intrathoracic, abdominal, or pelvic lymphadenopathy was identified, and there was no radiological evidence of an underlying malignancy.

Given the chronic constitutional symptoms, imaging findings, and epidemiological background, tuberculous EN was considered the leading diagnosis. The patient subsequently underwent ultrasound-guided aspiration of the collection, yielding approximately 35 mL of frank yellow purulent fluid. Placement of a drain was attempted but was unsuccessful because of the shallow nature of the collection. Samples were sent for routine bacterial and anaerobic cultures, fungal culture, *Actinomyces* testing, mycobacterial culture, and tuberculosis polymerase chain reaction (PCR) testing.

Gram staining demonstrated white blood cells but no organisms. Routine bacterial culture showed no growth after 48 hours, no anaerobic organisms were isolated after 10 days, and no fungi or yeasts were isolated after 14 days. Pleural fluid cytology demonstrated a paucicellular specimen comprising predominantly debris with scattered inflammatory cells. No granulomata or malignant cells were identified. No convincing mesothelial cells were present, and the specimen was therefore considered potentially non-representative. No sputum or other respiratory microbiological results were available for review, and no chest wall or rib tissue biopsy was performed.

Acid-fast bacilli were not identified on initial microscopy; however, mycobacterial culture subsequently became positive after seven days. PCR testing detected *M. tuberculosis* complex, with no mutations associated with rifampicin resistance detected. Whole-genome sequencing identified a lineage 1 (East-African-Indian-Ocean) *M. tuberculosis* isolate, with genotypic analysis predicting susceptibility to isoniazid, rifampicin, ethambutol, and pyrazinamide. However, the laboratory advised caution regarding the predicted pyrazinamide susceptibility. No phenotypic drug susceptibility results were documented in the available laboratory report. A diagnosis of tuberculous EN was therefore confirmed.

The patient was managed jointly by the respiratory, infectious diseases, microbiology, and tuberculosis specialist nursing teams. His recorded weight was 73.7 kg shortly before treatment initiation. Four-drug antituberculous therapy was commenced using Voractiv, five tablets once daily, with each tablet containing rifampicin 150 mg, isoniazid 75 mg, pyrazinamide 400 mg, and ethambutol 275 mg. This corresponded to total daily doses of rifampicin 750 mg, isoniazid 375 mg, pyrazinamide 2,000 mg, and ethambutol 1,375 mg. Pyridoxine 10 mg once daily was prescribed concurrently. Following the initial two-month intensive phase, the patient was transitioned to isoniazid and rifampicin for a further four months, with specialist review scheduled at the end of the planned six-month treatment course.

At the two-month follow-up, chest radiography demonstrated significant interval improvement in the previously noted right-sided pleural and parenchymal abnormalities following antituberculous treatment (Figures 3A, 3B).

**Figure 3:**
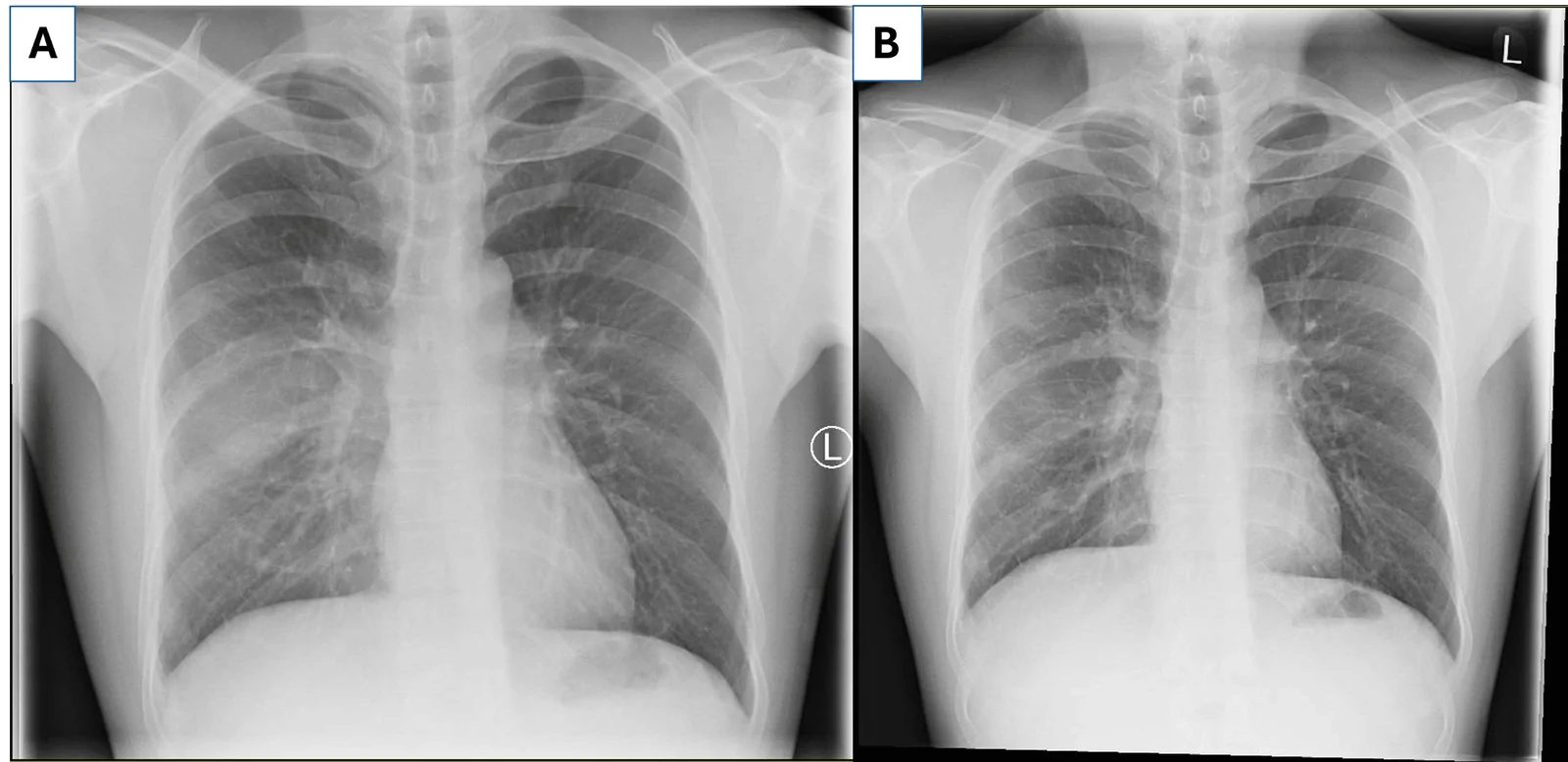
Chest radiographs before treatment and at two-month follow-up Posteroanterior chest radiographs obtained (A) before initiation of antituberculous treatment and (B) at two-month follow-up. The pretreatment radiograph demonstrates right perihilar and mid-zone opacification, with significant interval improvement following treatment.

## Discussion

Tuberculous EN can present insidiously, with clinical manifestations ranging from pleuritic chest pain and cough to constitutional symptoms and a gradually enlarging chest wall mass [2,3]. Similar presentations have been described in immunocompetent individuals, emphasizing that the absence of overt immunosuppression does not exclude the diagnosis [2,5]. In our patient, intermittent right-sided chest pain had been present for approximately two years, with worsening pleuritic pain, persistent dry cough, and night sweats preceding admission. Five months earlier, he had been treated for presumed right-sided pneumonia complicated by a pleural effusion; however, persistent radiographic abnormalities remained. The subsequent development of a tender, fluctuant posterior chest wall swelling was an important clinical clue to extension of the underlying pleural process.

Cross-sectional imaging is central to the diagnosis of EN. CT can demonstrate direct communication between a pleural collection and an extra-pleural soft-tissue component and assess extension into adjacent musculature and osseous structures [2,4]. In our patient, CT demonstrated a right posterior pleural collection extending through the chest wall into the adjacent paraspinal musculature, together with periosteal reaction of the adjacent ribs. Rib involvement has previously been reported in tuberculous EN and may occur through chronic contiguous spread of infection, occasionally resulting in tuberculous osteomyelitis [5].

Microbiological confirmation of pleural tuberculosis can be challenging because conventional tuberculous pleural effusions are frequently paucibacillary, resulting in limited diagnostic yield from pleural fluid microscopy and culture [6,7]. Molecular nucleic acid amplification tests can provide more rapid detection of *M. tuberculosis* and have high specificity, although their sensitivity in pleural fluid is variable; therefore, a negative molecular test does not reliably exclude pleural tuberculosis [6,7]. In our patient, acid-fast bacilli were not identified on initial microscopy; however, mycobacterial culture subsequently became positive after seven days, while targeted PCR testing detected *M. tuberculosis* complex. Whole-genome sequencing subsequently identified a lineage 1 East-African-Indian-Ocean isolate with predicted susceptibility to first-line antituberculous agents. This demonstrates the importance of specifically requesting mycobacterial investigations when tuberculosis is clinically suspected despite negative routine cultures.

Management of tuberculous EN requires appropriate antituberculous therapy together with consideration of drainage of the pleural or extra-pleural collection. Reported interventions include image-guided aspiration, intercostal drainage, video-assisted thoracoscopic surgery, decortication, and surgical debridement in selected patients with extensive chest wall or osseous involvement [3,5,8,9]. The need for invasive intervention should be individualized according to the size and accessibility of the collection, extent of surrounding tissue involvement, clinical condition, and response to treatment. In our patient, ultrasound-guided aspiration provided diagnostic material, although drain insertion was unsuccessful because of the shallow nature of the collection. As he remained clinically stable, standard four-drug antituberculous therapy with isoniazid, rifampicin, pyrazinamide, and ethambutol was commenced with multidisciplinary specialist follow-up.

This case highlights the importance of considering tuberculous EN in patients presenting with chronic constitutional symptoms and an unexplained chest wall swelling, even in the absence of recognized tuberculosis exposure or significant physiological disturbance. Persistent radiological abnormalities following treatment for presumed pneumonia or pleural infection should prompt reconsideration of the underlying diagnosis. Early cross-sectional imaging, aspiration of accessible collections, targeted mycobacterial testing, and multidisciplinary management can facilitate timely recognition and treatment of this uncommon manifestation of tuberculosis.

## Conclusions

Tuberculous EN is a rare but important complication of pleural tuberculosis that may present insidiously with chronic constitutional symptoms and a chest wall swelling. This case demonstrates the importance of maintaining a high index of suspicion when radiological abnormalities persist despite treatment for presumed pneumonia or pleural infection. CT is valuable in defining extension beyond the pleural space, while targeted mycobacterial testing can establish the diagnosis even when routine cultures are negative. Early recognition, multidisciplinary assessment, and appropriate antituberculous therapy are essential to achieving a favorable outcome and avoiding unnecessary diagnostic delay.
